# Machine learning-driven prognostic analysis of cuproptosis and disulfidptosis-related lncRNAs in clear cell renal cell carcinoma: a step towards precision oncology

**DOI:** 10.1186/s40001-024-01763-1

**Published:** 2024-03-16

**Authors:** Ronghui Chen, Jun Wu, Yinwei Che, Yuzhuo Jiao, Huashan Sun, Yinuo Zhao, Pingping Chen, Lingxin Meng, Tao Zhao

**Affiliations:** 1 School of Clinical Medicine, Shandong Second Medical University, Weifang, 261053 China; 2 Department of Oncology, People’s Hospital of Rizhao, Rizhao, 276826 China; 3 Department of Central Laboratory, Shandong Provincial Key Medical and Health Laboratory, Rizhao Key Laboratory of Basic Research on Anesthesia and Respiratory Intensive Care, The People’s Hospital of Rizhao, Rizhao, 276826 Shandong China; 4 Department of Pathology, People’s Hospital of Rizhao, Rizhao, 276826 China

**Keywords:** Clear cell renal cell carcinoma, Prognostic risk model, Machine learning algorithm, Cuproptosis, Disulfidptosis, Long non-coding RNA, Targeted drugs, Immune inhibitors

## Abstract

Cuproptosis and disulfidptosis, recently discovered mechanisms of cell death, have demonstrated that differential expression of key genes and long non-coding RNAs (lncRNAs) profoundly influences tumor development and affects their drug sensitivity. Clear cell renal cell carcinoma (ccRCC), the most common subtype of kidney cancer, presently lacks research utilizing cuproptosis and disulfidptosis-related lncRNAs (CDRLRs) as prognostic markers. In this study, we analyzed RNA-seq data, clinical information, and mutation data from The Cancer Genome Atlas (TCGA) on ccRCC and cross-referenced it with known cuproptosis and disulfidptosis-related genes (CDRGs). Using the LASSO machine learning algorithm, we identified four CDRLRs—ACVR2B-AS1, AC095055.1, AL161782.1, and MANEA-DT—that are strongly associated with prognosis and used them to construct a prognostic risk model. To verify the model's reliability and validate these four CDRLRs as significant prognostic factors, we performed dataset grouping validation, followed by RT-qPCR and external database validation for differential expression and prognosis of CDRLRs in ccRCC. Gene function and pathway analysis were conducted using Gene Ontology (GO) and Gene Set Enrichment Analysis (GSEA) for high- and low-risk groups. Additionally, we have analyzed the tumor mutation burden (TMB) and the immune microenvironment (TME), employing the oncoPredict and Immunophenoscore (IPS) algorithms to assess the sensitivity of diverse risk categories to targeted therapeutics and immunosuppressants. Our predominant objective is to refine prognostic predictions for patients with ccRCC and inform treatment decisions by conducting an exhaustive study on cuproptosis and disulfidptosis.

**Supplementary Information:**

The online version contains supplementary material available at 10.1186/s40001-024-01763-1.

## Introduction

Renal cancer is a common urologic malignancy, with 81,800 new cases of renal and pelvic cancer and 14,890 projected deaths according to the 2023 US cancer statistics [1]. Renal cell carcinoma (RCC) constitutes approximately 85% of all renal tumors, with clear cell renal cell carcinoma (ccRCC) being the most prevalent subtype, accounting for 75% of RCC cases [2, 3]. Surgical removal of renal tissue is the main treatment modality for early-stage ccRCC, and about 1/3 of ccRCC patients are already advanced at diagnosis, which often means high mortality, and metastasis rates [4]. ccRCC is characterized by a high degree of resistance to chemotherapy and a dense vascular distribution. Treatment commonly involves tyrosine kinase inhibitors (TKIs) targeting the VEGFR pathway [5]. Recent years have seen the validation of immunotherapy's efficacy, with studies indicating that combining TKIs with immunotherapy yields better clinical outcomes compared to monotherapy with targeted agents [6, 7]. Immune checkpoint inhibitors in combination with targeted agents are now the first-line option of choice for advanced ccRCC [8]. Prognostic scoring systems for ccRCC have contributed significantly to clinical diagnosis and prognostic assessment, for which better prognostic models are urgently needed to guide immune combination targeted drug therapy and to assess and guide the direction of clinical treatment for ccRCC patients.

The mechanism of action of antitumor drugs largely depends on inducing cell death, with numerous small molecules targeting cell death pathways having been identified and utilized in clinical trials [9]. Unlike known cell death mechanisms such as apoptosis, autophagy, and ferroptosis, the emergence of cuproptosis and disulfideptosis provides new directions for understanding cell metabolism and the tumor microenvironment (TME), laying the foundation for novel tumor treatment strategies. Cuproptosis is defined as the process whereby copper ions induce proteotoxic stress. This occurs when copper ions bind to the lipid-acylated constituents of the tricarboxylic acid cycle (TCA), instigating lipid-acylated protein aggregation. This aggregation subsequently results in the downregulation of iron-sulfur (Fe-S) cluster proteins, culminating in proteotoxic stress [10]. Disulfidptosis is a condition in which abnormal expression of SLC7A11 under glucose starvation causes a decrease in NADPH that counteracts disulfide toxicity and inadequate reduction of disulfide cystine, resulting in disulfide accumulation that generates disulfide stress, induces the disulfide bond between actin and cytoskeletal proteins to be stripped from the cytoplasmic membrane, and ultimately leads to cell death [11]. The discovery of cuproptosis and disulfidptosis represents a significant advancement in the identification of novel metabolic regulatory mechanisms in cancer [12, 13], potentially indicating new pathways for cancer therapy.

Cuproptosis and disulfidptosis are mechanisms of cellular demise intricately linked to the progression of various oncological pathologies, potentially serving as pivotal determinants of cancer prognoses [13, 14]. Studies conducted by Bian et al. [15] have posited that genes implicated in copper-mediated cytotoxicity may serve as viable prognostic biomarkers for ccRCC. Furthermore, research by Yuan et al. [16] underscores the significance of these genes in forecasting ccRCC patient outcomes in response to immunotherapeutic and targeted treatment modalities. Complementarily, Liu et al. [11] have substantiated the occurrence of disulfidptosis within renal carcinoma cells and in vivo animal models, facilitated by targeted pharmacological interventions. Prognostic models currently in development, which incorporate disulfidptosis-associated genetic markers, are poised to enhance prognostic accuracy and inform drug response evaluations in clinical settings [17, 18]. Despite existing research integrating ferroptosis with cuproptosis [19], studies investigating their concurrent impact remain elusive. Considering the pronounced association of ccRCC with both cuproptosis and disulfidptosis, it is hypothesized that their amalgamation could unveil novel avenues for prognostic refinement and therapeutic approaches for afflicted individuals.

Long non-coding RNAs (lncRNAs) are involved in the regulation of tumor protein-coding gene expression through binding to chromatin-modifying proteins, transcription factors, miRNAs, etc. [20]. lncRNAs regulate mitochondrial dynamics such as the TCA cycle, synthesis of cytoplasmic biological precursors, and in ccRCC cells through metabolic reprogramming to regulate cancer cell malignant transformation and control cellular energy expression [21, 22]. Machine learning, an offshoot of artificial intelligence, refines prediction models to forecast individual survival outcomes using extensive prognostic parameters [23]. Notably, the LASSO and Cox regression algorithms excel in accuracy and effectiveness for survival prediction modeling [24, 25]. The integration of machine learning with LncRNA analysis now markedly enhances prognostic and drug sensitivity assessments in oncology [26, 27]. Motivated by these advancements, we propose to leverage machine learning in investigating LncRNAs pertinent to cuproptosis and ferroptosis in ccRCC.

**Fig. 1:**
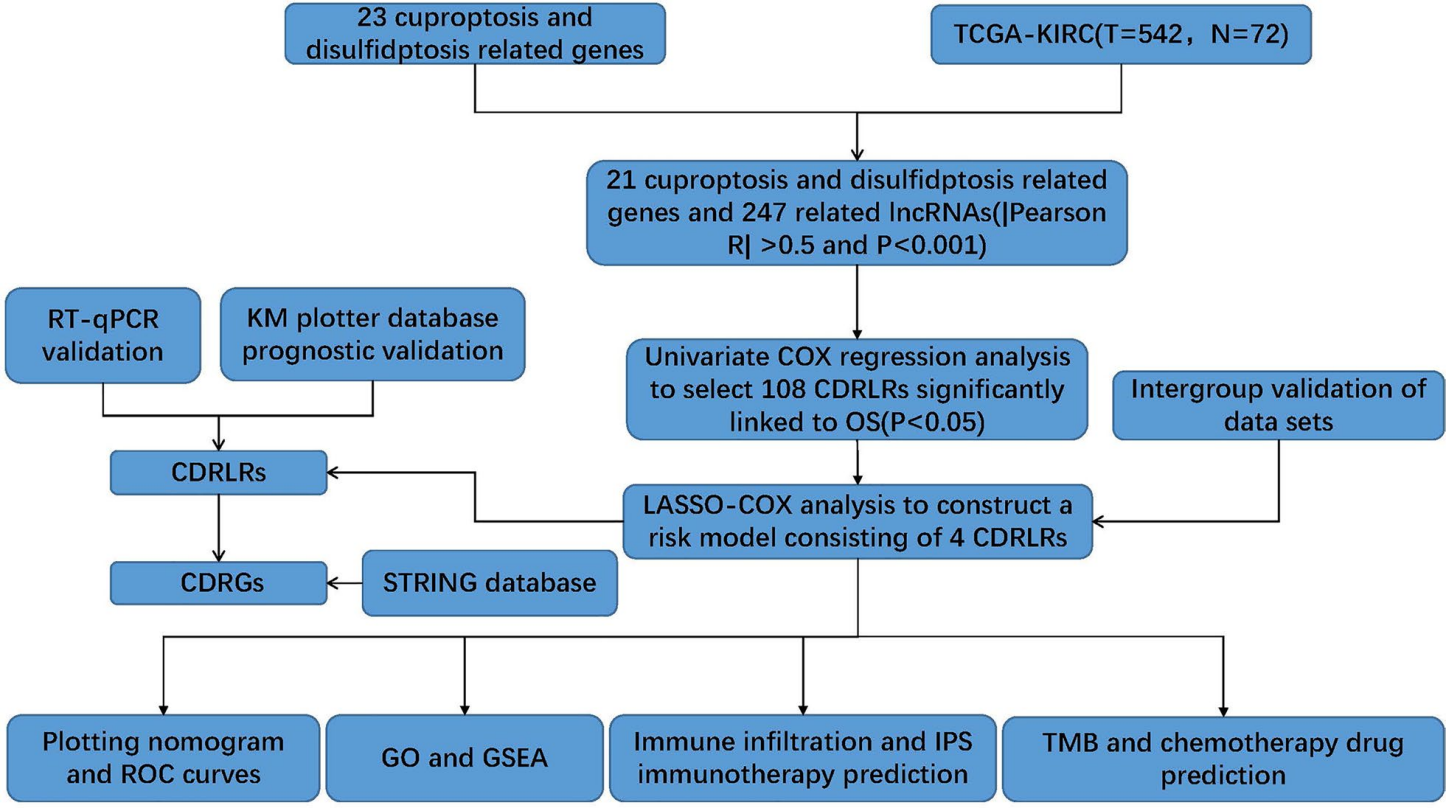
Research process flowchart

This study aims to develop a prognostic model of cuproptosis and disulfidptosis-related lncRNAs (CDRLRs) by analyzing real data of ccRCC patients in the TCGA database, combining cuproptosis and disulfidptosis-related genes, analyzing the TME, tumor mutational burden (TMB), and prognostic survival analysis to further analyze potential immune checkpoint inhibitors and targeted drugs with high clinical value. Figure 1 depicts the flowchart delineating the process of this study.

## Materials and methods

### Data collecting

The RNA-seq data for ccRCC were obtained from The Cancer Genome Atlas (TCGA) database (https://portal.gdc.cancer.gov) specifically the TCGA-KIRC dataset, which included details from 542 tumor samples and 72 normal tissue samples (accessed on 23 March 2023). The data were evaluated utilizing the “limma” package [28] in R software (version: R-4.2.2). This analysis incorporated pertinent clinical information including patient age, gender, tumor stage, histological grading, survival results, and duration of follow-up. To extract the RNA-seq data, we used a Perl script [29] (version Strawberry-perl-5.30.0.1; https://www.perl.org), and the extracted data were standardized to FPKM format. Genes with zero expression were excluded from the sample set. Additionally, somatic mutation data in mutation annotation format (MAF) were processed and visualized with the assistance of the “maftools” package [30].

### Differential expression identification and association of CDRGs with CDRLRs

We extracted genes related to cuproptosis and disulfidptosis-related genes (CDRGs) from various published sources [11, 31]. In total, 23 CDRGs were identified, with 13 being CDRGs: DLD, PDHB, ATP7B, ATP7A, DLAT, DLST, SLC31A1, DBT, FDX1, LIPIT1, LIAS, GCSH, and PDHA1, and the remaining 10 identified as disulfidptosis-related genes: GYS1, NDUFS1, OXSM, LRPPRC, NDUFA11, NUBPL, NCKAP1, RPN1, SLC3A2, and SLC7A11. The correlation between cuproptosis and disulfidptosis was analyzed based on the STRING database (https://cn.string-db.org/), and the network diagram of CDRGs was plotted using Cytoscape (version: 3.9.1). The expression matrix of cuproptosis and disulfidptosis-related lncRNAs was obtained using the R packages “BiocManager” and “limma” [28, 32], with selection criteria of |Pearson R|> 0.5 and p < 0.001. The Sankey diagram elucidating the relationship between CDRLRs and CDRGs was generated with the R packages “ggplot2” and “ggalluvial” [33].

### Construction and validation of risk model for CDRLRs by machine learning algorithms

LASSO represents a machine learning algorithm rooted in regression. It incorporates a regularization function atop logistic regression, mitigating overfitting and independently eliminating low correlation covariates to secure relatively substantial model variables. The LASSO-Cox regression algorithm, implemented via the “glmnet” R package, was utilized to scrutinize the correlation between CDRLRs and the overall survival (OS) of ccRCC patients. A comprehensive integration of univariate and multivariate Cox regression analyses enabled the identification of CDRLRs significantly related to OS. Subsequently, risk scores were computed for each patient using the ensuing formula:

$${\text{Risk Score}}={\sum }_{i=1}^{n}({\text{LncRNAexpi}}\times {\text{Coefi}})$$

The symbol “n” represents the quantity of ccRCC prognosis-associated CDRLRs, “i” symbolizes the ith CDRLR, and the expressions of lncRNA and regression coefficients are denoted by Coefi and LncRNAexpri respectively [34]. The training set, testing set, and the entire set were classified into high- and low-risk categories based on the median risk scores.

### Nomogram plotting and Kaplan–Meier (K–M) survival analysis

To measure the model’s accuracy, Receiver Operating Characteristic (ROC) curves accompanied by C-index plots were generated using R packages “survminer”, “survival”, “timeROC”, “rms” and “pec” [35]. The R package “regplot” was employed to produce nomogram and calibration plots, facilitating the prediction of patient prognosis and the assessment of prognostic accuracy. The correlation between clinical characteristics and Kaplan–Meier survival curves for high- and low-risk groups was delineated using the “survivor” and “survminer” R packages.

### GO and GSEA analysis

We applied the R package “clusterProfiler” [36] to perform Gene Ontology (GO) and Gene Set Enrichment Analysis (GSEA) on the differentially expressed genes (DEGs) within the high- and low-risk groups. We considered a P-value less than 0.05 to signify significant enrichment. We used “ggpubr” and “circlize” [37] to visualize the outcomes of the GO functional enrichment analysis, while we employed “enrichplot” to depict the results of the GSEA pathway analysis.

### TMB prognostic analysis and targeted drug prediction

Somatic mutation data of ccRCC patients were scrutinized employing the R package “maftools”. This facilitated the illustration of the somatic mutation landscape in both high- and low-risk groups, enabled the comparison of TMB variations between these groups, and provided further analysis within the context of patients’ prognosis. The R package “oncoPredict” [38] was used to predict the IC50 values of targeted therapeutics available for ccRCC within the high- and low-risk cohorts.

### TME analysis and immunotherapy prediction

The R packages “limma”, “ggpubr”, and “reshape2” were employed to construct violin plots with immune infiltration landscape maps for the high- and low-risk groups, thereby indicating the proportions of 22 types of tumor-infiltrating immune cells and comparing their differences. Furthermore, the R packages “limma”, “BiocManager”, “ggpubr", and “reshape2” were utilized to illustrate the disparities in immune function between the high- and low-risk groups. The study included an analysis of ccRCC patients regarding five immune checkpoint inhibitors: Programmed Death 1 (PD-1 or PDCD1), Programmed Death Ligand 1 (PD-L1 or CD274), Cytotoxic T Lymphocyte Antigen 4 (CTLA-4), Interleukin 6 (IL-6), and Lymphocyte Activating 3 (LAG3). The latter is a scoring scheme developed through machine learning algorithms to identify and quantify the determinants of tumor immunogenicity and has been demonstrated to predict solid cancers’ responses to CTLA-4 and PD-1 antibody-based immunotherapy [39]. The Cancer Immunome Atlas (TCIA, https://tcia.at/home) can be referred to for obtaining the Immunophenoscore (IPS) of ccRCC.

### Cell line culture and RT-qPCR

The human cell lines, 769-P and Caki-1, serve as specific models for ccRCC, whereas HK-2 functions as the normal control cell line. These cell lines are procured from Saibaikang, based in Shanghai, China. Each cell line was nurtured in a distinctive medium: HK-2 cells thrived in Dulbecco’s Modified Eagle Medium: Nutrient Mixture F-12 (DMEM/F12, Gibco, USA), 769-P in Roswell Park Memorial Institute 1640 medium (RPMI 1640, Gibco, USA), and Caki-1 in McCoy's 5A (Modified) Medium (McCoy's 5A, Gibco, USA). The media were fortified with 10% fetal bovine serum (FBS, Excell Bio, Uruguay) and 1% combined streptomycin and penicillin. Subsequently, the cells were incubated at 37 °C with 95% humidity in a dedicated cell incubator. Total RNA was isolated using TRIzol reagent (DP424, TIANGEN, Beijing, China) according to the manufacturer's instructions. cDNA synthesis was performed using total RNA using the PrimeScript RT kit (RR047A, TaKaRa, Beijing, China). Gene expression was quantified using SYBR Premix Ex TaqII (RR820A, TaKaRa, Beijing, China). All primers for RT-qPCR were synthesized by GENEWIZ Biotechnology Co., Ltd (Suzhou, China) (Additional file 1: Table S1). The PCR program was as follows: 40 cycles at 95 °C for 30 s, 95 °C for 5 s and 60 °C for 34 s. GAPDH was used as a standardized internal reference. Relative expression levels were estimated using the 2^−ΔΔCt^ method.

### External database validation and Statistical analysis

CDRLRs were validated using the Kaplan–Meier Plotter database [40] (accessed on 1st June 2023), a tool equipped for performing network survival analysis through both univariate and multivariate methods. Discrepancies in proportions of clinical characteristics were analyzed using the chi-square test. Differences between Kaplan–Meier curves were identified using the log-rank test. The analysis of PCR data was conducted using an independent sample *t*-test, facilitated by GraphPad Prism 8.0 software. A p-value less than 0.05 was deemed statistically significant.

## Results

### Construction of a prognostic risk model for ccRCC based on CDRLRs

**Fig. 2:**
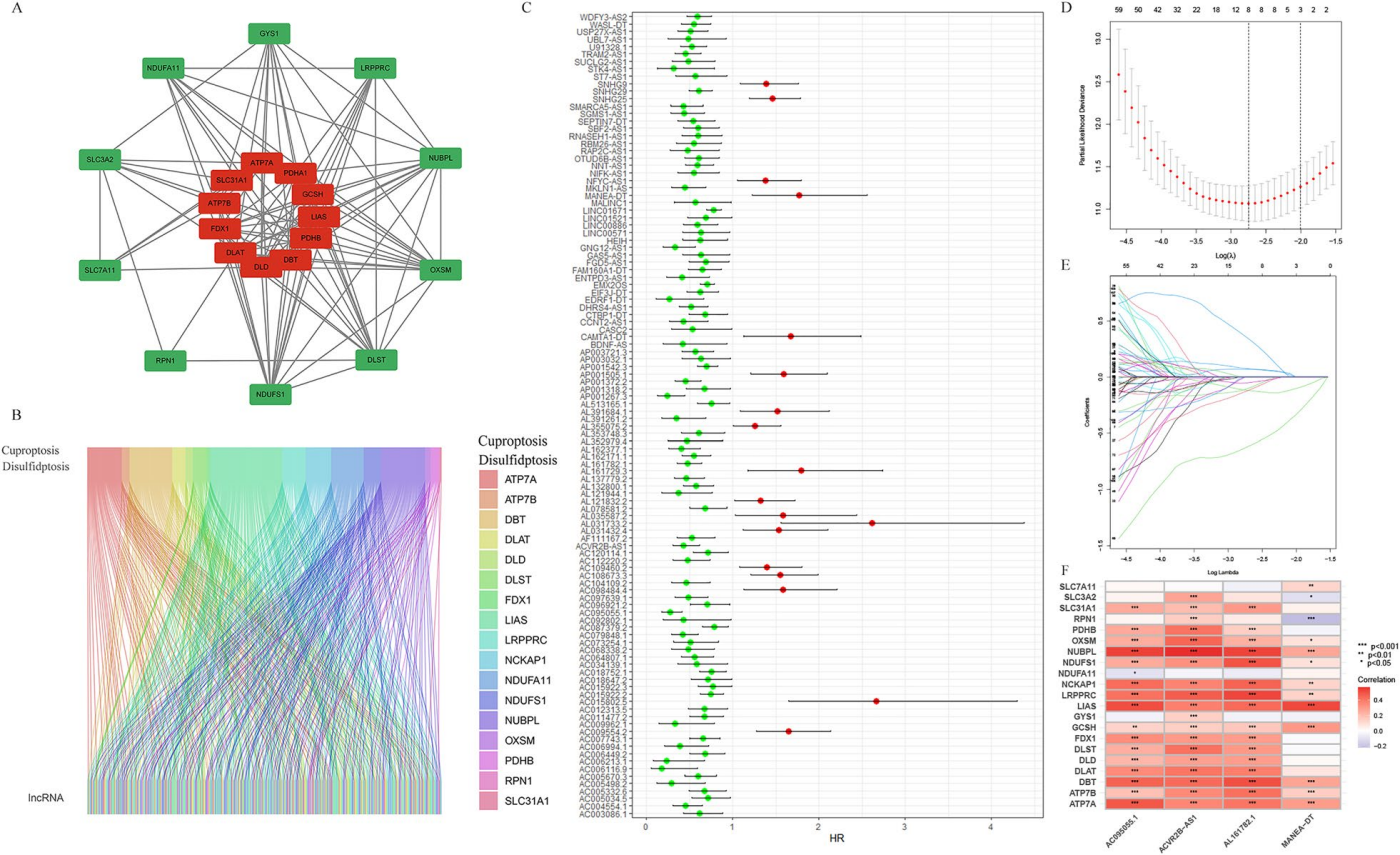
Identification of the prognostic CDRLRs and construction of prognostic risk model. **A** Protein–protein interactions among CDRGs based on STRING database. **B** The sankey relation between CDRGs and CDRLRs. **C** Forest plot showing univariate Cox regression analysis of prognosis-related CDRLRs (*p* < 0.05). **D** The LASSO regression coefficient spectrum. **E** Cross-validation of parameter selection in the LASSO model. **F** Heatmap of the correlation between CDRGs and 4 CDRLRs (*p < 0.05, **p < 0.01, ***p < 0.001)

Using the STRING database, we constructed a network relationship map of CDRGs (Fig. 2A) and demonstrated the association of cuproptosis and disulfidptosis death-related genes. In accordance with published literature and TCGA-KIRC data, 21 CDRGs were identified via co-expression analysis (Fig. 2B) (Additional file 1: Table S2). Following Pearson analysis, 247 eligible lncRNAs were established with parameters |Pearson R|> 0.5 and *p* < 0.001 (Additional file 1: Table S3). Univariate Cox regression analysis was applied to identify 108 lncRNAs significantly correlated with OS (*p* < 0.05, Fig. 2C). To prevent overfitting, LASSO regression was utilized, mitigating lncRNAs with a high correlation to prognosis. Subsequent multifactorial Cox regression resulted in the selection of four CDRLRs (Fig. 2D, E). The risk models were constructed using ACVR2B-AS1, AC095055.1, AL161782.1, and MANEA-DT (Additional file 1: Table S4). The corresponding risk score equations for ccRCC patients are provided below:

$${\text{Risk score }} = \, \left( { - 0.406602113479572 \times {\text{ExpACVR2B-AS1}}} \right) \, + \, \left( { - 0.988256841487476 \times {\text{ExpAC095055.1}} } \right) \, + \, \left( { - 0.526107034426687 \times {\text{ExpAL161782.1}}} \right) \, + \, \left( {0.988504048700137 \times {\text{ExpMANEA-DT}}} \right)$$

Subsequently, we generated a correlation heatmap to visualize the associations between four CDRLRs and CDRGs. This heatmap revealed that ten CDRGs—specifically, OXSM, NUBPL, NDUFS1, NCKAP1, LRPPRC, LIAS, GCSH, DBT, ATP7B, and ATP7A—showed a strong correlation with CDRLRs (Fig. 2F).

### Intergroup validation of prognostic risk models

**Fig. 3:**
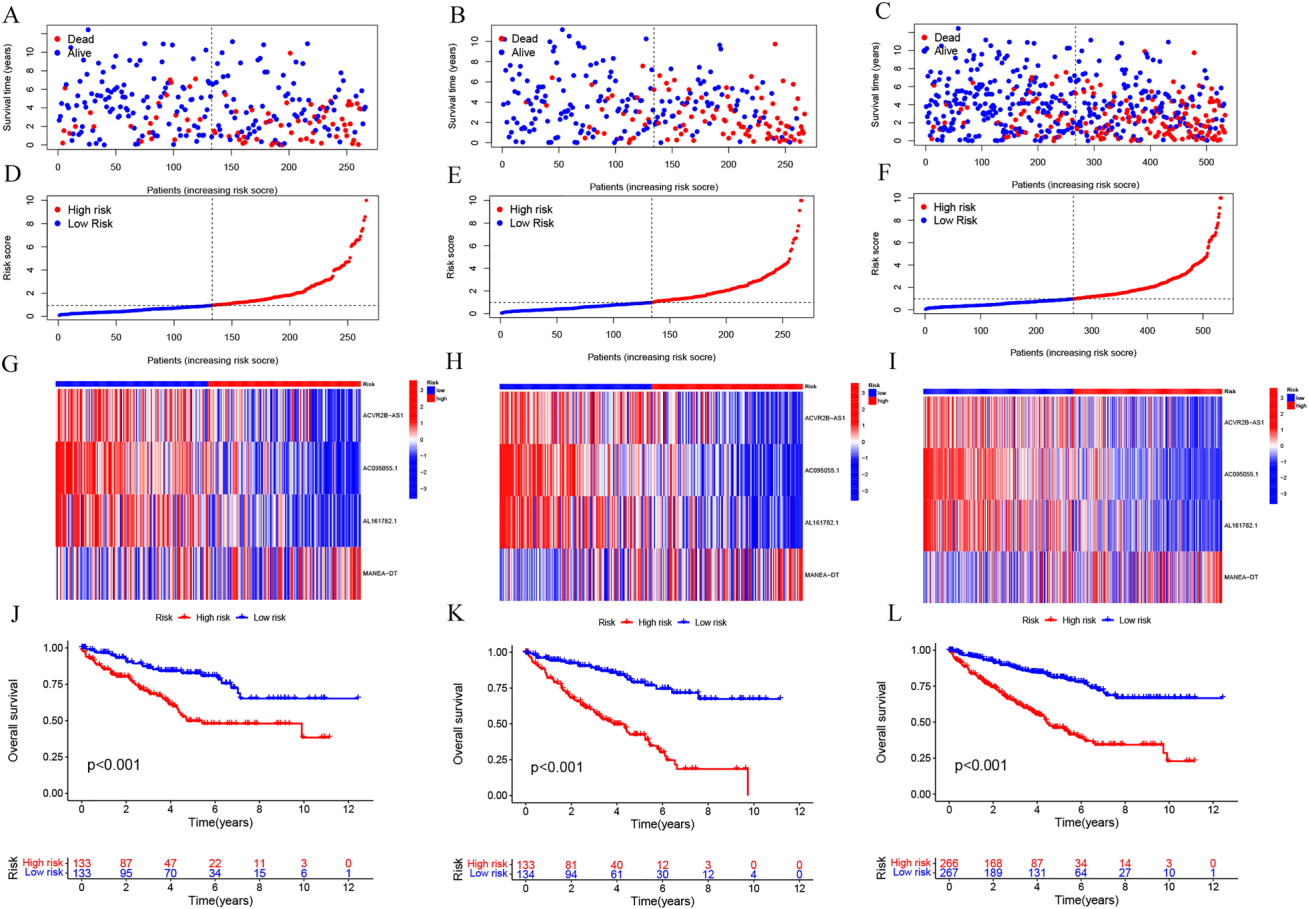
Validation of the prognostic risk model in the training, testing, and entire groups. **A**–**C** Survival status distribution maps. **D**–**F** Distribution of association between risk score and survival status between high- and low-risk groups. **G**–**I** Risk heatmap of the four CDRLRs. **J**–**L** Kaplan–Meier survival curves between high- and low-risk groups

Median risk scores were computed based on CDRLRs. Subsequently, the training set, test set, and the entire set were divided into high- and low-risk groups for survival analysis. This revealed an increasing mortality rate among ccRCC patients in correlation with escalating risk scores (Fig. 3A–F). In the low-risk group, ACVR2B-AS1, AC095055.1, and AL161782.1 exhibited significant expression, whereas MANEA-DT was highly expressed in the high-risk group (Fig. 3G–I). This reinforced that ACVR2B-AS1, AC095055.1, and AL161782.1 are beneficial prognostic factors and MANEA-DT is a poor prognostic factor. The high-risk group demonstrated significantly lower OS compared to the low-risk group (Fig. 3J–L). These findings were confirmed in the three data sets.

### Independent prognosis of risk scores

**Fig. 4:**
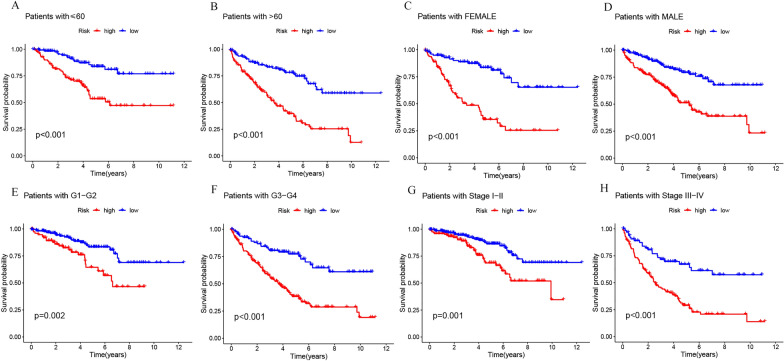
Kaplan–Meier survival curve analysis of high- and low-risk groups in ccRCC patients with different age (**A**, **B**), gender (**C**, **D**), histological grade (**E**, **F**), and tumor stage (**G**–**H**)

**Fig. 5:**
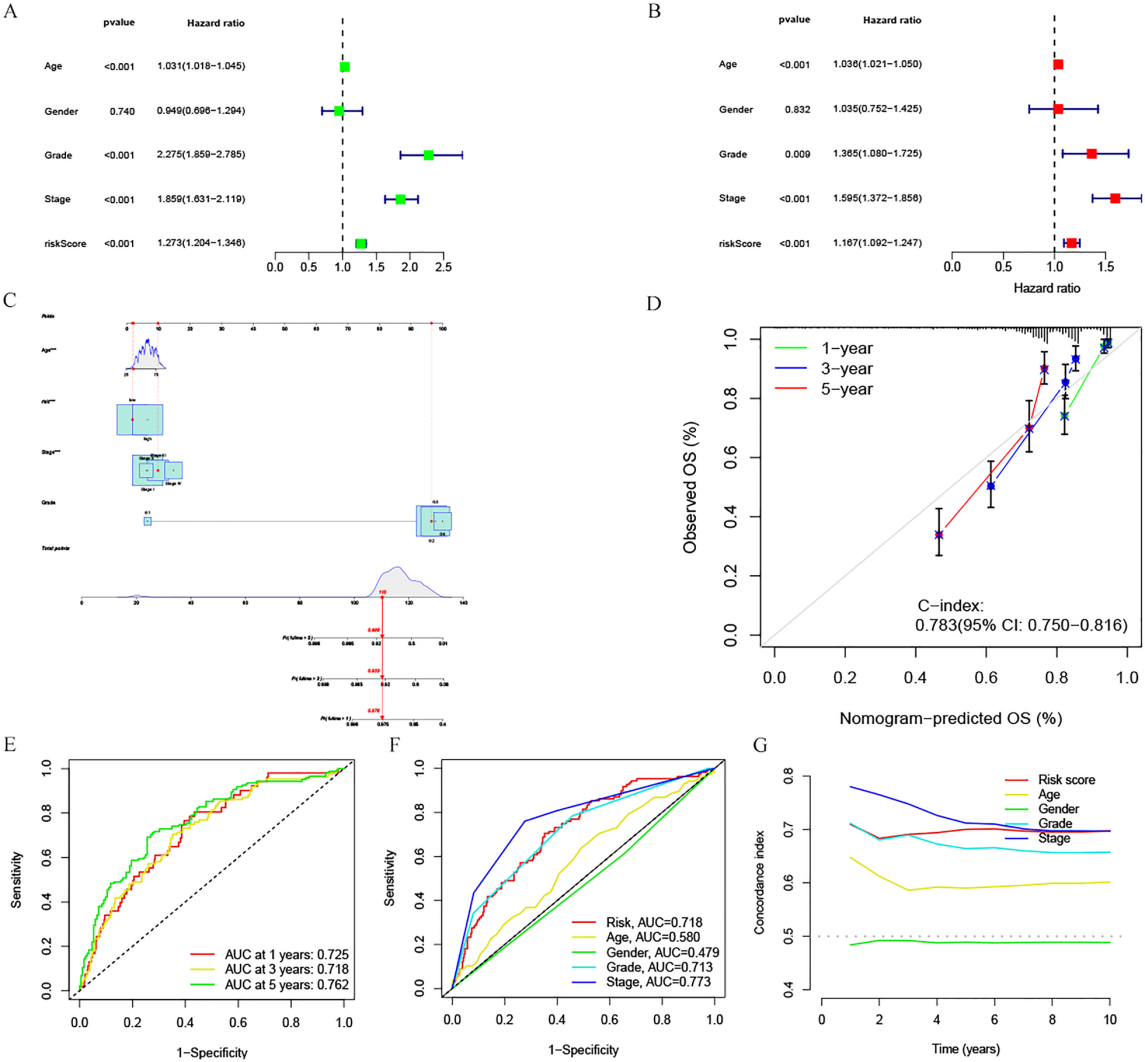
Independent prognostic value assessment of risk scores and clinical characteristics. **A** Univariate Cox regression analysis of risk scores and clinical characteristics. **B** Multivariate Cox regression analysis of risk scores and clinical characteristics. **C** Nomogram of 1, 3 and 5-year OS of ccRCC patients after excluding unrelated variables (*p < 0.05; **p < 0.01; ***p < 0.001). **D** Calibration curves for OS at 1, 3 and 5 years. **E** ROC validation curve for 1, 3 and 5 years-OS risk model in ccRCC patients. **F** Prognostic function comparison of risk model and other clinical characteristics. **G** Risk model and other clinical characteristics for 10-year concordance index

A comparison of survival probability among ccRCC patients in high- and low-risk groups, based on patient age, gender, histological grade, and tumor stage, revealed that the risk score effectively assessed prognosis across all these clinical characteristics (*p* < 0.01, Fig. 4A–H). Risk scores were indeed adept at predicting OS in ccRCC patients, independent of clinical characteristics. After univariate and multivariate Cox regression analyses, risk scores and clinical characteristics such as age, histological grade, and tumor stage emerged as independent prognostic factors in ccRCC patients (p < 0.01, Fig. 5A, B). Factors correlating negatively with prognosis were excluded, and nomogram plots were drawn based on independent prognostic factors (Fig. 5C). Calibration curves were subsequently used to verify the reliability of these findings, which revealed a C-index value of 0.783 (95% CI 0.7750–0.816) (Fig. 5D). The 1-year, 3-year, and 5-year subject operating characteristic curves (ROC) were then plotted, with an area under the curve (AUC) of 0.725, 0.718, and 0.762, respectively (Fig. 5E). When the AUC values of clinical characteristics within each group were compared, the risk score’s AUC was 0.718, second only to tumor stage (Fig. 5F). The 10-year Concordance index further validated these findings (Fig. 5G). These results suggest that the risk score surpasses other clinical characteristics, except tumor stage, as a factor in assessing prognosis. Thus, the risk score can effectively serve as a biomarker for predicting ccRCC patient prognosis.

### GO and GSEA of high- and low-risk groups

**Fig. 6:**
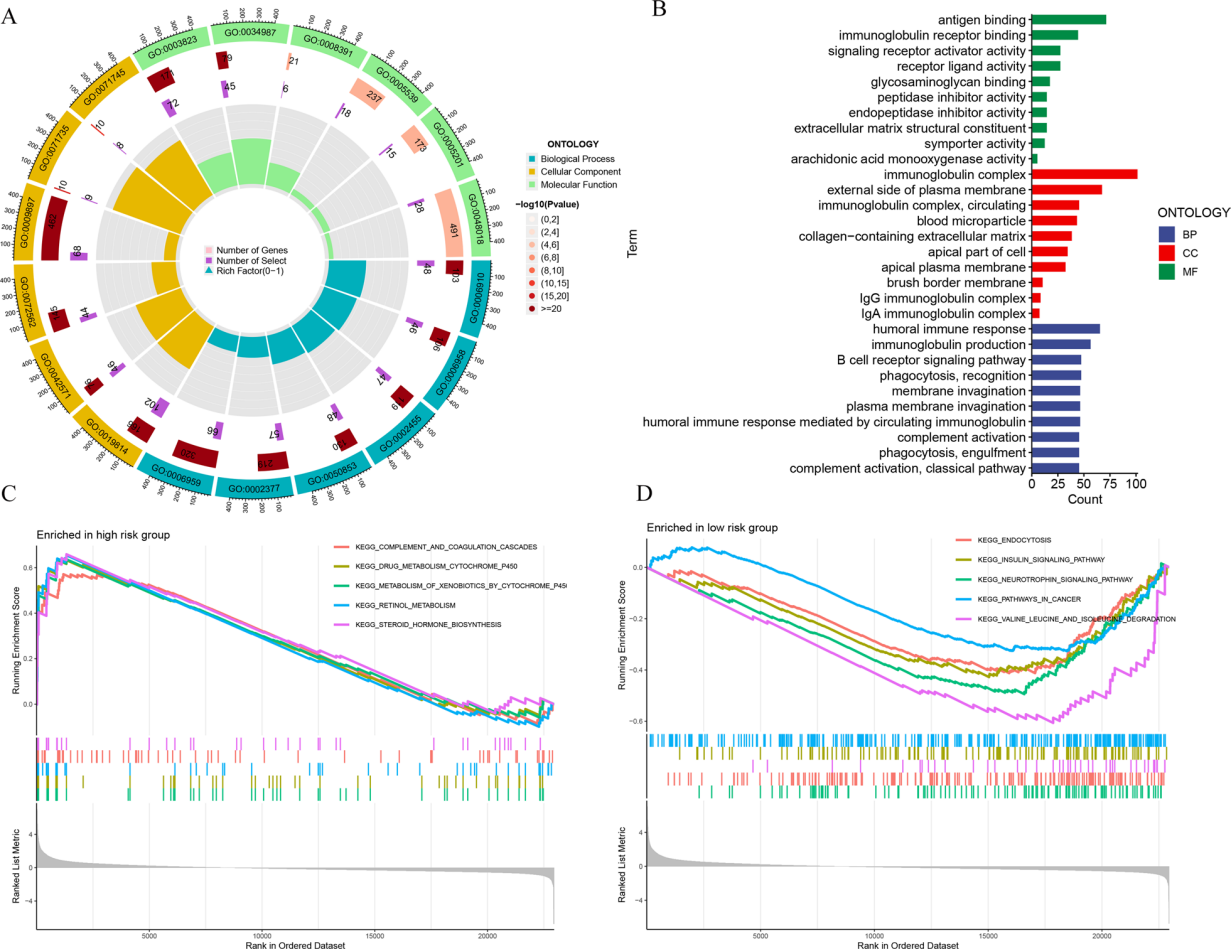
Identification and analysis of the biological functions and pathways of DEGs of CDRGs. GO enrichment analysis of **A** circle and **B** bar graphs showing the BP, CC, and MF groups of DEGs. **C**, **D** GSEA plots significantly enriched top five pathways between high- and low-risk groups

From the high- and low-risk groups, we identified 683 DEGs that met the selection criteria (Padjust < 0.05, |log2 (fold change)|≥ 1) (Additional file 1: Table S5). These DEGs were used for functional and pathway enrichment analyses to explore potential biological differences between the groups (Fig. 6A). The Gene Ontology (GO) enrichment analysis showed an enrichment of biological processes (BP), such as antigen binding and immunoglobulin receptor binding. Cellular components (CC) involving the immunoglobulin complex and the external side of the plasma membrane were also enriched, along with molecular functions (MF) like humoral immune response and immunoglobulin production (Fig. 6B) (Additional file 1: Table S6). Utilizing Gene Set Enrichment Analysis (GSEA), we identified differences in pathways between the risk groups, with 89 signaling pathways significantly enriched (*p* < 0.05) (Additional file 1: Table S7). The high-risk group displayed significant enrichment in the top five pathways: complement and coagulation cascades, drug metabolism by cytochrome P450, metabolism of xenobiotics by cytochrome P450, retinol metabolism, and steroid hormone biosynthesis (Fig. 6C). Conversely, the low-risk group exhibited enrichment in the following top five pathways: endocytosis, insulin signaling pathway, neurotrophin signaling pathway, pathways in cancer, and valine leucine and isoleucine degradation (Fig. 6D). These enrichment patterns may offer valuable insights into the prognostic differences observed between the high- and low-risk groups.

### Immune cell infiltration and immunotherapy sensitivity

**Fig. 7:**
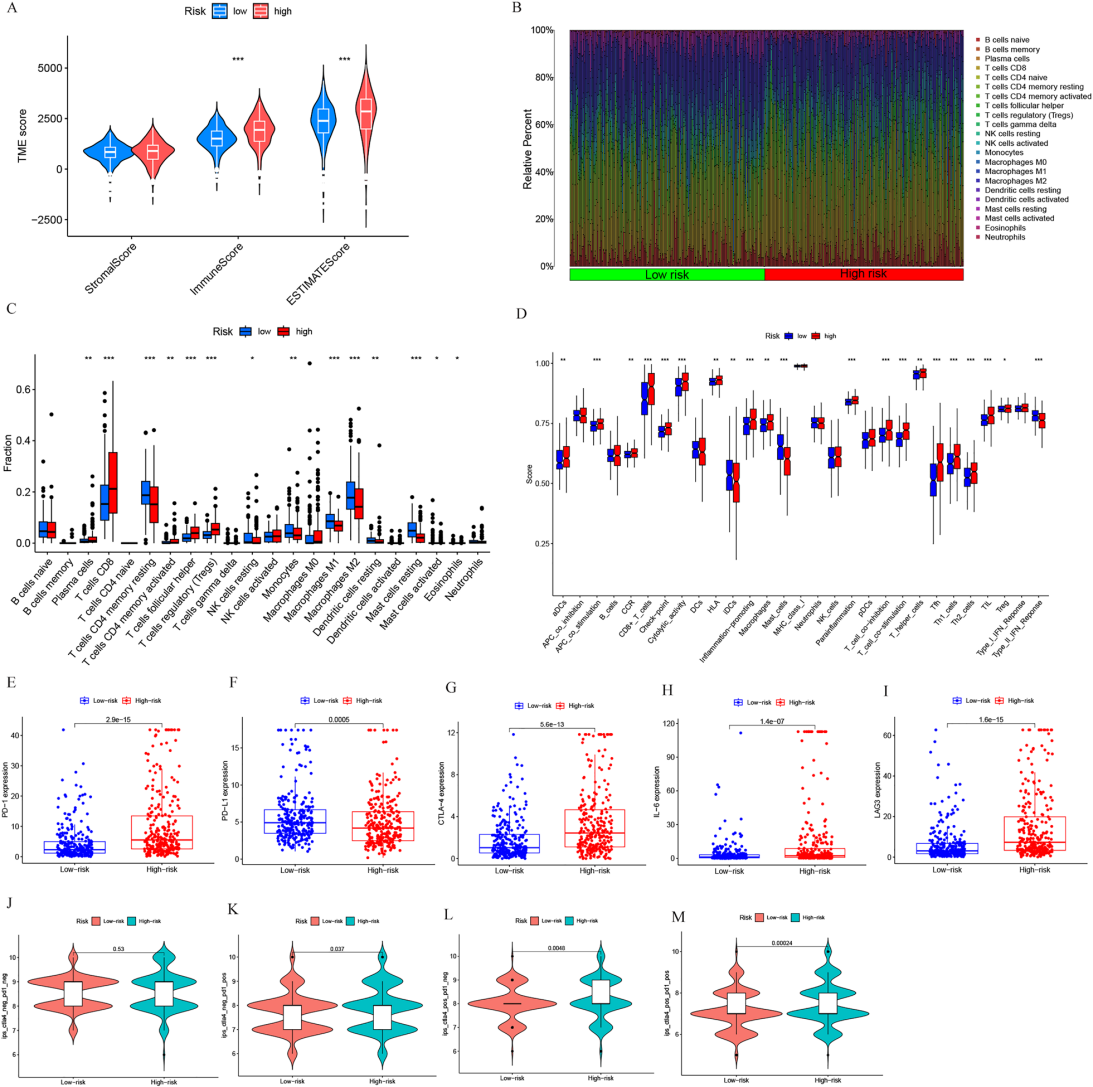
TME and sensitivity to immune checkpoint inhibitors analysis of high- and low-risk groups. **A** Association of high- and low-risk groups with stromal, immune, and ESTIMATE algorithm scores (***p < 0.001). **B** Immune cell infiltration landscape. **C** Differences in immune cell infiltration between high- and low-risk groups. **D** Differences in immune function scores between high- and low-risk groups. Boxplot of PD1 (**E**), PD-L1 (**F**), CTLA-4 (**G**), IL-6 (**H**), and LAGE (**I**) expression in different risk groups. Four types of IPS classification based on risk score grouping [CTLA4-neg-PD1-neg (**J**), CTLA4-neg-PD1-pos (**K**), CTLA4-pos-PD1-neg (**L**), and CTLA4-pos-PD1-pos (**M**)]

Significant differences were observed in TME scores, notably ESTIMATE scores (*p* < 0.001) and immune scores (*p* < 0.001), among ccRCC patients, with the high-risk group presenting notably higher scores than the low-risk group (Fig. 7A). To explore potential relationships in immune cell infiltration between the risk groups, we compared 22 immune cell enrichment scores and 29 immune-related function enrichment scores (Additional file 1: Tables S8 and S9). Using the CIBERSORT algorithm, we created an immune infiltration landscape for the high- and low-risk groups. The correlation box line plot illustrated significant associations between multiple immune cells and risk scores (Fig. 7B). High-risk groups showed enrichment of T cells CD8, T cells follicular helper, and T cells regulatory (Tregs) (*p* < 0.001), while the low-risk group exhibited a significant upregulation of T cells CD4 memory resting, Macrophages M1, Macrophages M2, and Mast cells resting (*p* < 0.001, Fig. 7C). Our immune function analysis indicated that the risk models demonstrated significant discrepancies across multiple immune function scores, including the checkpoint (*p* < 0.001, Fig. 7D). Guided by these immune function analyses, we compared the differential expression of five key immune checkpoints (PD1, PD-L1, CTLA-4, IL-6, LAG3) using the IPS. Results showed that except for PD-L1, which was highly expressed in the low-risk group, all other checkpoints were overexpressed in the high-risk group (*p* < 0.001, Fig. 7E–I). This suggests the IPS's potential in predicting the immune response to checkpoint inhibitors in ccRCC patients based on risk score grouping. The immune efficacy, predicted by PD-1 and CTLA-4 expression in the TCIA database, yielded significantly different risk scores in the ctla4(−) pd1( +), ctla4( +) pd1(−), and ctla4( +) pd1( +) groups (*p* < 0.05). Higher scores were found in the high-risk group, indicating that high-risk ccRCC patients demonstrated a heightened sensitivity to PD-1 and CTLA-4 single-agent and dual-agent combination immunotherapies (Fig. 7J–M).

### TMB prognostic analysis and potential drug sensitivity

**Fig. 8:**
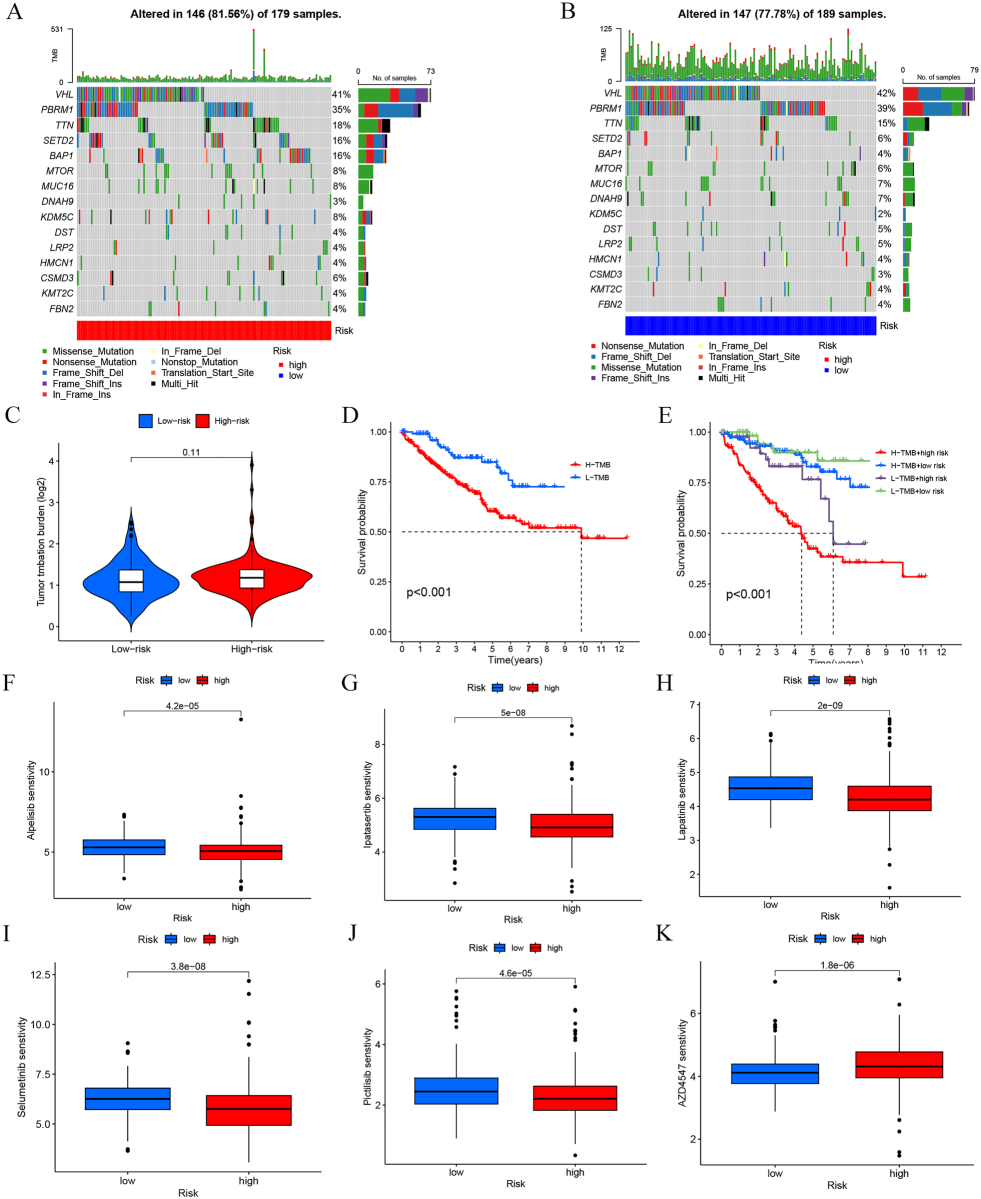
TMB and sensitivity to targeted drugs analysis of high- and low-risk groups. **A** Genes with the top 20 mutation frequencies in the high-risk group. **B** Genes with the top 20 mutation frequencies in the low-risk group. **C** Boxplot of TMB and risk group correlations. **D** The relationship between TMB and Kaplan–Meier survival. **E** Kaplan–Meier survival with TMB status and risk level. Differences in drug sensitivity across risk groups for **F** Alpelisib, **G** Ipatasertib, **H** Lapatinib, **I** Selumetinib, **J** Pictilisib, and **K** AZD4547

To explore somatic mutations within the high- and low-risk groups, TCGA-KIRC mutation data were downloaded and categorized. The results demonstrated identical 15 driver genes with the highest mutation frequencies in both groups, with STED2 and BAP1 hypermutations being more prevalent in the high-risk group (Fig. 8A, B). Although no statistically significant association was found between the risk groups and TMB (*p* = 0.11, Fig. 8C), both TMB grouping and TMB + risk grouping significantly differentiated survival statuses of ccRCC patients (*p* < 0.001, Fig. 8D, E). Here, the High-TMB + high risk group exhibited the lowest overall survival rate, while the Low-TMB + low risk group showed the highest. Thus, a combination of the risk score and TMB presents a promising prognostic marker for patients. Several common drugs were selected to analyze their sensitivity in the risk groups. The results indicated that Alpelisib, Ipatasertib, Lapatinib, Selumetinib, and Pictilisib demonstrated higher sensitivity in the low-risk group, whereas AZD4547 showed high sensitivity in the high-risk group (*p* < 0.0001, Fig. 8F–K).

### Differential expression and prognostic validation of CDRLRs in ccRCC

**Fig. 9:**
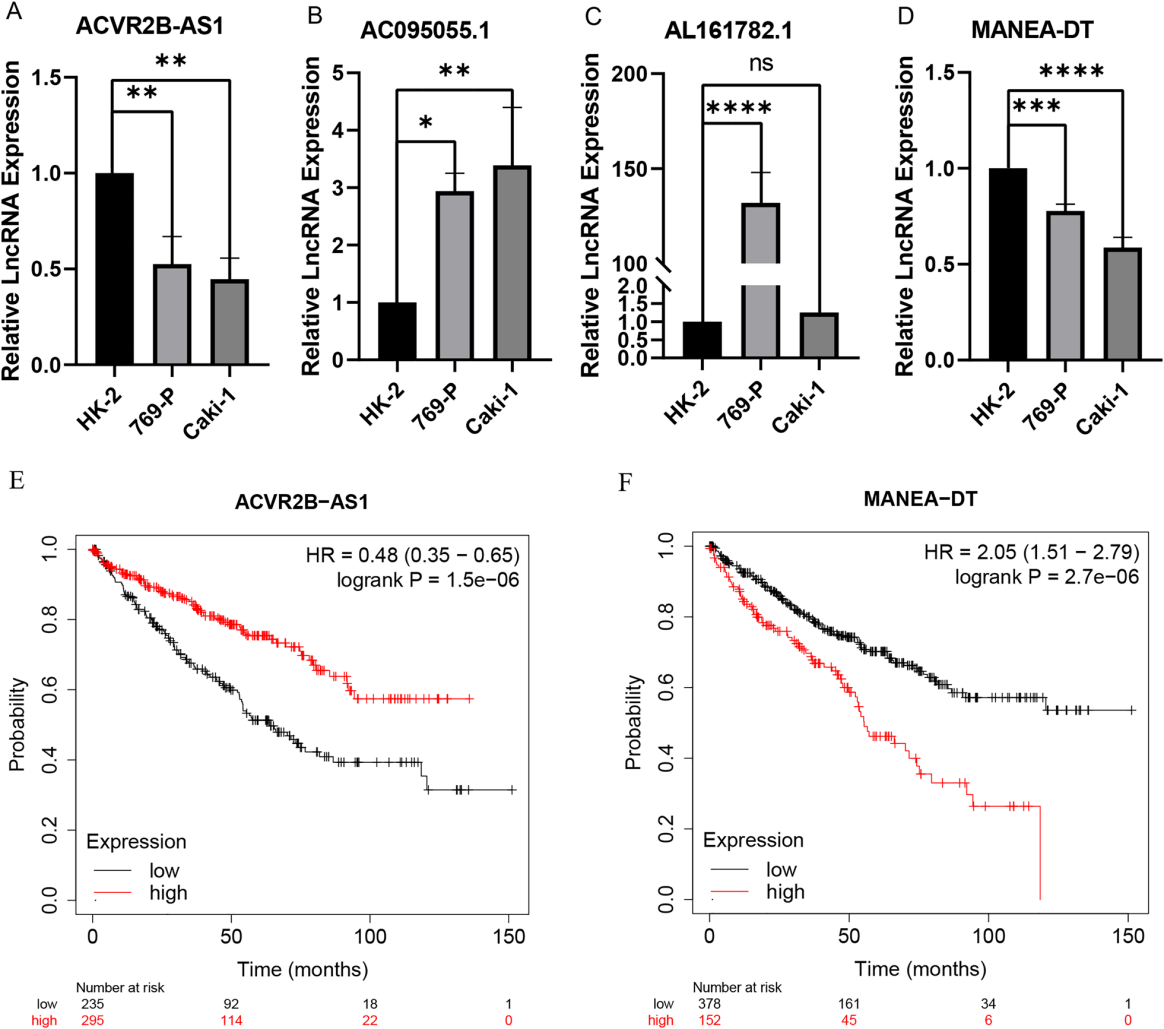
Validation of CDRLRs in cell lines and prognostic validation of external database. **A**–**D** RT-qPCR validation of CDRLRs expression levels in normal and clear cell renal cell carcinoma cell lines and expression levels of four CDRLRs in HK-2, 769-P, and Caki-1 cells (*p < 0.05; **p < 0.01; ***p < 0.001; ****p < 0.0001). **E**, **F** OS analysis of ACVR2B-AS1 and MANEA-DT from the Kaplan–Meier Plotter database

To further examine CDRLRs' expression in ccRCC, we utilized two ccRCC cell lines (769-P, Caki-1), with normal renal tubular epithelial cells (HK-2) as a control. RT-qPCR evaluated the mRNA expression levels of the four key CDRLRs in these cell lines. The findings demonstrated a significantly higher expression of AC095055.1 in both 769-P (*p* < 0.05) and Caki-1 (*p* < 0.01) cell lines compared to the HK-2 cell line (Fig. 9B). However, AL161782.1 showed significant expression only in the 769-P cell line (*p* < 0.0001, Fig. 9C). Conversely, ACVR2B-AS1 (*p* < 0.01) and MANEA-DT (*p* < 0.0001) expressions were significantly lower in 769-P and Caki-1 cell lines compared to HK-2 (Fig. 9A, D). To corroborate the independent prognostic role of CDRLRs in ccRCC patients, we performed a prognostic analysis of ACR2B-AS1 and MANEA-DT using the KM Plotter database (AC095055.1 and AL161782.1 were not found in the database). The results identified ACR2B-AS1 as a protective prognostic factor (HR = 0.48 (0.35–0.65), *p* < 0.0001), while MANEA-DT (HR = 2.05 (1.51–2.79), *p* < 0.0001) indicated a poor prognosis (Fig. 9E, F). This prognosis based on CDRLRs aligns with the survival analysis results from external databases.

## Discussion

ccRCC is the most common type of renal cell carcinoma, characterized by high heterogeneity, frequent recurrence, a significant risk of metastasis, and a poor prognosis [41]. Approximately 30% of patients present with advanced metastasis at the time of diagnosis [42], and the disease often shows resistance to both radiotherapy [43, 44] and systemic treatments, which significantly impacts patient survival and prognosis. Recent advances in immunotherapy and targeted therapeutic mechanisms have revolutionized treatment options for ccRCC [45]. ccRCC is a malignant tumor characterized by high immune infiltration and dense vascular distribution [46, 47], Regulating specific metabolic pathways in renal cancer, including ccRCC, can modulate immune cell responses and inflammatory characteristics [48–51]. Immune cells within the TME can inhibit tumor growth by eliminating cancer cells but may also protect certain cancer cell subpopulations, contributing to drug resistance [52–54]. Recent research has shown that activating or inhibiting cuproptosis-related metabolic pathways can alter the TME composition of ccRCC, thereby impacting the overall treatment response [55, 56]. Additionally, models of renal cancer subtypes based on disulfidptosis, utilizing transcriptomic analysis and characterization of immune infiltration, demonstrate the potential of disulfidptosis biomarkers in predicting the efficacy of targeted and immunotherapeutic drugs [57]. However, given the abundance of biometric sequencing data for tumor patients and the complexity of clinical characteristics, manual analysis is insufficient for discerning underlying correlations. Machine learning algorithms, as an innovative approach within the field of artificial intelligence, have revolutionized the analysis of large datasets, offering promising prospects in advancing the subtype analysis, mechanistic research, and treatment strategies for renal cancer [58, 59]. Han et al. [60] have differentiated renal cancer subtypes using radiographic imaging combined with machine learning algorithms to assess prognosis. Similarly, Li et al. [61] explored the association between radiomics models and VHL gene mutation characteristics to understand their correlation further. Previous studies have used machine learning to investigate ferroptosis-related lncRNAs and build prognostic models for ccRCC patients [62]. Bai et al. [26] and Zhao et al. [63] have similarly employed machine learning to study copper-related lncRNAs and disulfide-related genes, respectively, and construct survival models for ccRCC and bladder cancer. These models provide valuable guidelines for cancer immunotherapy and targeted therapy. Currently, the development of cancer prognostic models that integrate the analysis of ferroptosis, cuproptosis, and disulfidptosis is at the forefront of oncological research, aiming to predict drug sensitivity and validate crucial factors through in vitro experimentation [64, 65]. In this study, we utilized a machine learning algorithm, LASSO regression, to construct a risk-prognosis model based on CDRLRs. We then combined this risk score with TMB and TME for further analysis, to assess targeted drug sensitivity. Additionally, by applying an immune immunophenoscore algorithm, we evaluated the susceptibility to immune checkpoint inhibitors, offering significant contributions to the therapeutic direction of ccRCC and addressing the existing gap in CDRLR-related research.

Copper ions, which are integral to human physiological processes as cofactors for key metabolic enzymes, are regulated by a network of copper-dependent proteins to maintain low levels within the body [66]. Notably, tumor tissues and serological copper ion levels are typically higher in cancer patients than in normal controls. While dysregulation of copper homeostasis may trigger cytotoxic responses, altered copper ion levels can also influence cancer development [67]. Cancer cells often show preferential induction in copper carriers, and the application of copper ion carriers and copper chelators in anticancer therapy has shown promise for inducing cancer cell death by modulating copper metabolism [68]. ccRCC is characterized by metabolic reprogramming. Wettersten et al. [69] revealed hierarchical metabolic reprogramming in ccRCC tumor tissues through proteomics and metabolomics. Studies have shown that the regulation of the TCA cycle and fatty acid synthesis correlates with tumor aggressiveness and survival rates in ccRCC patients [70, 71]. Cuproptosis, a cell death mechanism characterized by protein lipoylation in the TCA cycle [72], has been shown by Nanni et al. [73] to offer cancer preventive effects through controlling metabolic extracts related to copper ion regulation, affecting mitochondrial and DNA damage pathways linked to cuproptosis. Genes associated with cuproptosis have been identified as potential predictors for prognosis, immunotherapy, and targeted therapy in ccRCC patients [15, 74]. Disulfidptosis results from metabolic reactions caused by the toxicity of disulfide bonds and the accumulation of cysteine under conditions of glucose starvation following SLC7A11 dysfunction [11]. SLC7A11, a key protein in regulating cancer cell metabolism, allows most cancer cells to intake cystine, reduce it to cysteine, and then use it for glutathione synthesis for antioxidation. Yan et al. [75] discovered that overexpression of SLC7A11 can modulate oxidative stress in metastatic cancer cells and inhibit tumor metastasis. Notably, 90% of ccRCC cases involve the loss of both alleles of the VHL gene [76], and VHL-deficient ccRCC exhibits a specific dependency on cysteine for glutathione synthesis, making it a therapeutic target. Activating the Src-p38 signaling pathway leads to cysteine-deprivation-induced necrosis, but this mechanism becomes inactive upon VHL restoration [77]. While the FDA has approved targeted agents such as sunitinib for ccRCC treatment, these agents carry significant cytotoxicity [78]. Belzutifan, approved by the FDA for immunotherapy in renal cell carcinoma associated with VHL syndrome [79], can also be used in combination with the multi-targeted anticancer agent cabozantinib for immunotherapy-resistant clear cell renal carcinoma [80]. Even though immune inhibitors have been widely applied in ccRCC patients, their potential for drug resistance warrants significant attention [81]. Consequently, cuproptosis and disulfidptosis exhibit immunogenic properties that regulate immune cell infiltration and modulate tumor metabolism. Extensive research has demonstrated a potential association between these two cell death mechanisms and the prognosis and drug resistance in ccRCC patients. A comprehensive and in-depth investigation of cuproptosis and disulfidptosis within ccRCC patients could enhance prognostic predictions and aid in circumventing specific drug resistance mechanisms.

In our research, we leveraged the STRING database to clarify the relationship between cuproptosis and disulfidptosis. We then employed machine learning algorithms to investigate the correlation between the clinical prognosis of ccRCC patients in the TCGA database and CDRLRs. The culmination of our study was the development of a prognostic model anchored on ACRR2B-AS1, AC095055.1, AL161782.1, and MANEA-DT. ACRR2B-AS1 is a long non-protein coding RNA. Recent studies have identified ACVR2B-AS1 as an OS-independent prognostic factor in digestive system tumors, including gastric and hepatocellular carcinomas, leading to the construction of prognostic models based on this lncRNA [82, 83]. Our study is the first to confirm the independent prognostic significance of ACVR2B-AS1 in ccRCC. AL161782.1 has been utilized in creating relevant prognostic models for m6A and cuproptosis mechanisms in ccRCC-related studies. However, comprehensive studies on two lncRNAs, MANEA-DT and AC095055.1, remain scarce. Our research pioneers the validation of ACRR2B-AS1 and MANEA-DT as independent prognostic factors for ccRCC through RT-qPCR experiments at the mRNA level using cell lines, complemented with data from external databases.

The cuproptosis and disulfidptosis-related risk scores displayed superior prognostic evaluation capability when compared to other clinical attributes, exhibiting 1-, 3- and 5-year AUC values exceeding 0.7. This highlights the robust reliability of risk scores in predicting the prognosis of ccRCC patients. The high-risk group, classified based on median risk score, was notable for high CD8 + T cell infiltration, significant mutations in the BAP1 gene, and worse prognosis compared to the low-risk group. These observations largely align with the subtype group characteristics noted in prior studies [47, 84, 85]. Immune function scores revealed a substantial increase in immune checkpoint function scores within the high-risk group compared to the low-risk group. Consequently, significant expression differences in five crucial immune checkpoints between high and low-risk groups were confirmed. The high-risk group demonstrated high expression in all immune checkpoints except CD274, suggesting an increased sensitivity to immunotherapy. Further analysis of the immunophenoscore of these targets revealed a notable response in ccRCC patients to combined PD-1 and CTLA-4 immune therapies. This indicates that dual-target immunotherapy in ccRCC patients, based on risk score assessments, may be beneficial. The prognostic superiority of combined PD-1 and CTLA-4 inhibitors as a first-line treatment for advanced RCC has been clinically validated [86, 87]. Although clinical data on immunotherapy's prognostic influence in ccRCC patients are sparse, it provides valuable direction for subsequent clinical trials. In the context of targeted drug sensitivity, the PI3K inhibitor Alpelisib was subjected to screening. Rugo et al. [88] observed a significant reduction in adverse effects and improved progression-free survival with Alpelisib in conjunction with the fulvestrant regimen. Though Curigliano et al. [89] found minimal changes in the pharmacokinetics of Alpelisib combined with everolimus ± exemestane for renal cell carcinoma, our model presents novel insights for the application of Alpelisib. Ipatasertib, an Akt inhibitor, in combination with chemotherapy, or Lapatinib, an EGFR/HER-2 tyrosine kinase inhibitor, has shown efficacy. Ipatasertib, an Akt inhibitor, has demonstrated good tolerability and safety when combined with chemotherapy or hormonal treatments for prostate cancer, though its effect on renal cell carcinoma remains unexplored [90, 91] Lapatinib, an EGFR/HER-2 tyrosine kinase inhibitor, exhibits better overall tolerance compared to hormonal therapy among advanced RCC patients who have progressed following initial cytokine treatment. Moreover, it offers survival benefits to EGFR (3 +) patients. Selumetinib, a selective MEK1 inhibitor, coupled with Everolimus, can attenuate angiogenesis during renal cell carcinoma proliferation by reducing VEGF secretion, consequently enhancing antitumor activity [92]. Rausch et al. [93] achieved a significant reduction in cell line metabolic activity and induction of apoptosis in renal cell carcinoma utilizing optimized low-dose combinations of AZD4547 (an FGFR signaling pathway inhibitor) and pictilisib (a pan-phosphatidylinositol 3-kinase inhibitor). Notably, distinct sensitivity differences between AZD4547 and pictilisib have been observed across different risk groups, underscoring the need for in-depth investigation into the mechanisms of cuproptosis and disulfidptosis.

Our study is subject to several limitations. Firstly, there is an inherent bias in the transcriptomics and clinical data available in public databases, necessitating the support of additional external clinical trial data to strengthen our retrospective analysis. Secondly, our investigation into the genes associated with cuproptosis and disulfidptosis lacks empirical experiments to verify the mechanisms and functional relationships. Lastly, our research only extends to external validation of differential expression for the lncRNAs involved in our model at the cellular and protein levels. In the future, we aim to enhance the generalizability and reliability of our model through integrative approaches such as radiomics and histopathomics, among other multi-omics studies.

## Conclusions

In this study, we devised a cuproptosis and disulfidptosis-related prognostic risk model for ccRCC patients using machine learning algorithms. The reliability of four CDRLRs as key prognostic factors was corroborated through external databases and experiments. This process further facilitated the prediction of the sensitivity to immune and targeted drugs and implied guidance for prognostic assessment and personalized treatment for ccRCC patients.

### Supplementary Information

**Additional file 1: Table S1.** Primers for RT-qPCR. **Table S2.** The expression matrix of cuproptosis and disulfidptosis-related genes (CDRGs). **Table S3.** The expression matrix of cuproptosis and disulfidptosis-related lncRNAs (CDRLRs). **Table S4.** The risk scores for all samples based on four key CDRLRs. **Table S5.** DEGs between high- and low-risk groups. **Table S6.** Detailed information of GO analysis for high- and low-risk groups. **Table S7.** Detailed information of GSEA analysis for high- and low-risk groups. **Table S8.** The proportion of immune cells in all samples according to CIBERSORT algorithm. **Table S9.** Immune function score of all samples.

## Data Availability

The data supporting the findings and conclusions of this study are available in the article.
